# Evidence architecture of glaucoma-related biomaterials reveals an uneven transition toward smart materials, additive manufacturing, and functional tissue engineering

**DOI:** 10.3389/fbioe.2026.1868297

**Published:** 2026-06-12

**Authors:** Jiabin Wang, Tianyi Sui, Yabo Ma, Yang Sui, Zhe Qin, Shipeng Wang, Haojun Ma, Wenxin Xue, Daijiang Wang

**Affiliations:** 1 Senior Department of Ophthalmology, Chinese PLA General Hospital, Beijing, China; 2 Medical School of Chinese PLA, Beijing, China

**Keywords:** additive manufacturing, biomaterials, functional tissue engineering, glaucoma, hydrogel, retinal ganglion cell, smart materials, trabecular meshwork

## Abstract

**Background:**

Glaucoma-related biomaterial research has expanded from ocular drug delivery to responsive hydrogels, anti-fibrotic systems, glaucoma drainage device (GDD) and minimally invasive glaucoma surgery (MIGS)-related interfaces, retinal ganglion cell protection, trabecular meshwork models, and additive manufacturing. Whether this expansion represents a coherent transition toward smart, manufacturable, and function-oriented tissue-engineering systems remains unclear.

**Methods:**

We conducted an AI-assisted, rule-guided, and manually audited evidence architecture reconstruction of glaucoma-related biomaterial studies published from 2006 to 2025. Records from Web of Science Core Collection, Scopus, and PubMed were integrated, deduplicated, parsed from RIS files, screened, and quality controlled. Retained studies were classified by application scenario, evidence level, and translational features. Core evidence records were assigned to five operational levels, from material preparation and physicochemical characterization to disease-microenvironment intervention, long-term functional integration, and clinical or advanced translational evidence. Theme maturity and the convergence of smart material, additive manufacturing, and tissue-engineering relevance were further assessed.

**Results:**

From 1,227 parsed records, 596 were retained, including 547 core evidence studies and 49 review or background records. In the core evidence set, Level 1 to Level 5 evidence included 93, 57, 140, 99, and 158 records, respectively. Level 1–3 evidence accounted for 53.0% of the core evidence set, whereas Level 4–5 evidence accounted for 47.0%, indicating a substantial but unevenly distributed translational component. *In situ* hydrogels and contact lens-based delivery systems represented the largest application categories, whereas retinal ganglion cell protection, trabecular meshwork modeling, anti-fibrosis after glaucoma surgery, GDD/MIGS-related interfaces, and 3D printing represented smaller but more disease-specific or integration-oriented domains.

**Conclusion:**

Glaucoma-related biomaterials are moving beyond passive delivery platforms, but their transition toward smart materials, additive manufacturing, and functional tissue engineering remains uneven. Future studies should emphasize reproducible material design, disease-relevant functional endpoints, outflow-pathway models, neuroprotection, and engineered surgical interfaces.

## 1 Introduction

Glaucoma is a chronic neurodegenerative eye disease characterized by progressive retinal ganglion cell loss, optic nerve damage, and irreversible visual field deterioration. Although intraocular pressure remains the only established modifiable risk factor, long-term disease control is limited by poor adherence to topical therapy, insufficient ocular bioavailability, postoperative fibrosis, device-related tissue response, and the lack of clinically relevant models for aqueous outflow dysfunction (Berdahl et al., 2024; Fu et al., 2024). These problems cannot be fully addressed by pharmacology alone. They also involve drug residence, material–tissue interfaces, wound-healing regulation, neural protection, and disease-relevant model construction. In this sense, glaucoma provides a demanding but informative setting for evaluating whether biomaterials can move beyond passive delivery and contribute to functional therapeutic systems.

Biomaterial strategies for glaucoma were initially dominated by ocular drug delivery, including hydrogels, *in situ* gels, nano- and microparticles, contact lenses, implants, and sustained-release formulations designed to improve drug retention and reduce dosing burden (Dang et al., 2022; Chawnani et al., 2024). More recent studies have begun to extend this framework. Responsive hydrogels, anti-fibrotic matrices, bioactive coatings, glaucoma drainage device (GDD)- and minimally invasive glaucoma surgery (MIGS)-related interfaces, retinal ganglion cell-protective systems, trabecular meshwork models, and 3D printed constructs now appear across the literature (Li et al., 2021). This shift is consistent with broader developments in smart materials and additive manufacturing, where material systems are expected not only to carry therapeutic agents, but also to respond to local cues, support controlled release, guide cellular behavior, and enable reproducible construction of tissue-relevant platforms (Fea et al., 2022).

Despite this expansion, the field remains difficult to judge from publication counts or material categories alone. A large number of studies may still remain at the stage of formulation development, physicochemical characterization, *in vitro* release, cytotoxicity testing, or short-term animal validation. Such evidence is necessary, but it does not necessarily indicate disease-modifying activity or translational maturity. Conversely, smaller areas such as anti-fibrosis after glaucoma surgery, trabecular meshwork modeling, retinal ganglion cell protection, GDD/MIGS interface engineering, and additive manufacturing may contain fewer publications but stronger links to disease mechanisms or functional endpoints. Existing reviews have summarized material types and delivery strategies, while conventional bibliometric analyses mainly describe publication trends, keywords, journals, or collaboration networks (Kim and Lee, 2024; Xu et al., 2025). What remains unclear is how the evidence is distributed across experimental depth, disease relevance, and translational readiness.

Here, we reconstructed the evidence architecture of glaucoma-related biomaterial research from 2006 to 2025. Instead of treating the literature as a collection of publication counts, we organized retained records according to three linked questions. First, we classified core studies by operational evidence level to determine how far each record progressed from material preparation to disease-relevant or translational validation. Second, we compared major application themes to identify high-volume delivery areas, smaller disease-facing domains, and emerging translational niches. Third, we assessed the overlap among smart material relevance, additive manufacturing relevance, and tissue-engineering relevance to determine whether these trajectories had begun to converge in glaucoma-related material research. Through this framework, the study identifies not only where the field has expanded, but also where evidence maturity and functional integration remain uneven.

## 2 Materials and methods

### 2.1 Study design

This study was designed as an evidence architecture reconstruction of glaucoma-related biomaterial research published from 2006 to 2025. The analysis focused on how the literature was organized across disease application scenarios, experimental depth, translational maturity, and its relationship to smart materials, additive manufacturing, and functional tissue engineering.

**FIGURE 1:**
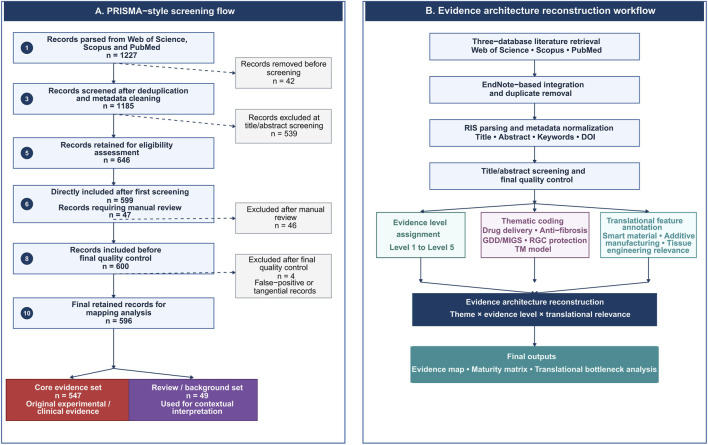
Study selection and evidence architecture reconstruction workflow. **(A)** PRISMA-style flow diagram showing record identification, screening, eligibility assessment, manual review, final quality control, and separation of retained records into the core evidence set and review/background set. **(B)** Analytical workflow for evidence architecture reconstruction, including three-database retrieval, EndNote-based integration, RIS parsing, metadata normalization, evidence-level assignment, thematic coding, translational feature annotation, and final evidence mapping.

The workflow included literature retrieval, cross-database integration, duplicate removal, RIS-based metadata parsing, title and abstract screening, eligibility assessment, evidence-level classification, thematic coding, translational feature annotation, and visualization. A rule-guided framework was used for classification, with AI-assisted text screening applied during record processing and manual audit applied to ambiguous or boundary cases. In this study, AI-assisted processing was used as a screening and text-structuring aid rather than as an autonomous decision-maker. It supported preliminary identification of candidate glaucoma-related material records, recognition of relevant terms in titles, abstracts, and keywords, and flagging of records requiring manual review. Final inclusion, exclusion, evidence-level assignment, theme classification, and adjudication of borderline cases were determined according to predefined operational rules and manual audit. Review and background articles were retained for contextual mapping but were not included in the core evidence-level analysis. The overall workflow is summarized in Figure 1.

### 2.2 Data sources and search strategy

Records were retrieved from Web of Science Core Collection, Scopus, and PubMed. The search covered publications from 2006 to 2025 and combined glaucoma-related terms with material-related terms.

The glaucoma-related terms included glaucoma, ocular hypertension, intraocular pressure, trabecular meshwork, retinal ganglion cell, RGC, optic nerve, trabeculectomy, glaucoma filtration surgery, glaucoma drainage device, and minimally invasive glaucoma surgery. The material-related terms included hydrogel, *in situ* gel, thermosensitive gel, stimuli-responsive material, smart material, biomaterial, polymer, scaffold, coating, implant, contact lens, drug delivery, controlled release, sustained release, nanoparticle, microparticle, microneedle, 3D printing, bioprinting, additive manufacturing, and tissue engineering.

The search was conducted in two steps. A main search was used to capture the broad glaucoma-related biomaterial literature. Sensitivity searches were then performed to improve retrieval of emerging or easily missed topics, including smart materials, stimuli-responsive systems, 3D printing, additive manufacturing, tissue engineering, organ-on-a-chip, trabecular meshwork models, and bioengineered outflow models. Search terms were adapted to the syntax of each database. The complete database-specific search strategies, including main and sensitivity searches for Web of Science Core Collection, Scopus, and PubMed, are provided in Supplementary Table S1. Database-level raw hit counts were reported only when directly available from the original search outputs and were not reconstructed retrospectively. The search strategy and screening documentation are summarized in Table 1.

**TABLE 1:** Corpus construction and screening summary.

Section	Item	Search date	Time span	n
Database search	Web of Science Core Collection - Main search	2026-04-26	2006-2025	-
Database search	Scopus - Main search	2026-04-26	2006-2025	-
Database search	PubMed - Main search	2026-04-26	2006-2025	-
Database search	Web of Science Core Collection - Sensitivity search	2026-04-26	2006-2025	-
Database search	Scopus - Sensitivity search	2026-04-26	2006-2025	-
Database search	PubMed - Sensitivity search	2026-04-26	2006-2025	-
Screening and eligibility	Records parsed from merged RIS file	-	-	1227
Screening and eligibility	Records after deduplication and metadata cleaning	-	-	1185
Screening and eligibility	Records excluded during title/abstract screening	-	-	539
Screening and eligibility	Records requiring manual review	-	-	47
Screening and eligibility	Records excluded after manual review	-	-	46
Screening and eligibility	Records retained before final quality control	-	-	600
Screening and eligibility	Records excluded after final quality control	-	-	4
Screening and eligibility	Final retained records for mapping analysis	-	-	596
Screening and eligibility	Core evidence records	-	-	547
Screening and eligibility	Review/background records	-	-	49

Database-level records should be completed using the original search log. Screening numbers were calculated from the final classified dataset.Database-level raw hit counts were reported only when verifiable from the original search log and were not reconstructed retrospectively. Complete database-specific search strategies are provided in Supplementary Table S1. Screening numbers were calculated from the final classified dataset.

### 2.3 Record integration, deduplication, and screening

Records exported from the three databases were imported into EndNote 21 for cross-database integration and preliminary duplicate removal. During data export testing, tab-delimited files from EndNote showed unstable field alignment and inconsistent placement of abstracts, keywords, and indexing terms. RIS files were therefore used for downstream parsing and metadata normalization.

Parsed records were organized into structured tabular files. Available metadata fields included title, abstract, keywords, publication year, journal, DOI, publication type, and database source. Deduplication was performed using DOI-based matching when DOI information was available, followed by exact or near-exact title matching for records without DOI. When duplicate records contained different levels of metadata completeness, the most complete record was retained as the primary entry.

Screening was performed at the title and abstract level. Records were considered eligible when they addressed a glaucoma-related disease context and involved a biomaterial, hydrogel, polymeric platform, nano/microparticle system, implant, coating, contact lens, scaffold, 3D printing strategy, tissue-engineering model, or related material-based intervention. Records were excluded when glaucoma was mentioned only incidentally, when the study did not involve material design or material-based intervention, or when ambiguous abbreviations did not refer to glaucoma-relevant concepts. The abbreviation “MIGS” was interpreted as minimally invasive glaucoma surgery only when supported by the title, abstract, keywords, or device context.

Records with insufficient abstracts but clear glaucoma-material relevance in the title, keywords, or publication context were flagged for manual review. Review and background articles were retained when they provided relevant context for glaucoma-related biomaterials, drug delivery devices, smart material systems, or surgical materials, but they were separated from primary evidence records during evidence-level analysis. The manual-review triggers, decision rules, and record-level outcomes are documented in Supplementary Table S2.

### 2.4 Analytical sets

Two analytical sets were defined before analysis. The retained set included all records judged relevant after screening and final quality control. This set was used for the overall literature landscape, annual trends, application scenario distribution, and convergence analysis of smart material, additive manufacturing, and tissue-engineering relevance.

The core evidence set included primary studies with experimental, translational, clinical, modeling, or material-validation content. This set was used for evidence-level classification, theme-specific evidence distribution, and thematic maturity analysis. Review and background articles were excluded from Level 1–5 evidence statistics to avoid mixing narrative synthesis with primary evidence.

### 2.5 Evidence-level classification

Core evidence records were assigned to one of five operational evidence levels according to the highest level of validation identifiable from the title, abstract, keywords, and available metadata. When a record contained features corresponding to more than one evidence level, it was assigned to the highest identifiable level supported by the available record content. Lower-level characterization or *in vitro* endpoints were therefore not counted separately once a higher validation level was present. The classification focused on experimental depth and translational maturity rather than citation count, journal impact, or publication type.

Level 1 referred to material preparation and physicochemical characterization, including material synthesis, gelation behavior, rheology, swelling, degradation, porosity, mechanical properties, printability, drug loading, or formulation characterization without cellular, animal, disease-functional, or clinical validation.

Level 2 referred to *in vitro* release or cell-based validation, including drug release, permeability testing, cytotoxicity, cell viability, cellular uptake, inflammatory markers, apoptosis markers, or assays involving trabecular meshwork cells, retinal ganglion cells, corneal epithelial cells, fibroblasts, or other relevant cell types, without animal or clinical validation.

Level 3 referred to conventional animal model validation, including short-term ocular residence, ocular pharmacokinetics, ocular tolerability, short-term intraocular pressure reduction, basic histology, and general biocompatibility in animal models, without deeper disease-microenvironment intervention, long-term integration, or tissue-engineering functional endpoints.

Level 4 referred to disease microenvironment intervention or tissue-engineering mechanism validation, including post-surgical fibrosis, scar modulation, inflammation, oxidative stress, retinal ganglion cell protection, trabecular meshwork or aqueous outflow modeling, glaucoma drainage device or minimally invasive glaucoma surgery-related material interfaces, tissue remodeling, or other glaucoma-relevant functional endpoints.

Level 5 referred to advanced translational, long-term functional, or clinical evidence, including clinical or human studies, long-term follow-up, large-animal validation, long-term implantation performance, sustained intraocular pressure control, tissue integration, long-term neuroprotection, or visual-function-related endpoints.

The operational definitions and classification boundaries for each evidence level are provided in Table 2.

**TABLE 2:** Evidence-level classification rules.

Evidence level	Definition	Operational criteria	Typical endpoints	Included in core evidence analysis	Example keywords
Level 1	Material design, fabrication or physicochemical characterization without biological disease validation	Studies were assigned to level 1 when the main evidence was limited to material synthesis, gelation behavior, rheology, swelling, degradation, morphology, printability, drug loading capacity or other physicochemical measurements, without cell, animal or clinical validation relevant to glaucoma	Rheology; gelation time; swelling ratio; degradation; porosity; mechanical strength; printability; drug loading efficiency	Yes	hydrogel preparation; rheology; swelling; degradation; printability; material characterization
Level 2	*In vitro* drug release or cellular validation without *in vivo* glaucoma-relevant functional testing	Studies were assigned to level 2 when they reported *in vitro* release profiles, permeability assays, cytotoxicity, ocular cell compatibility, or mechanistic cell experiments, but did not provide animal or clinical evidence of intraocular pressure control, anti-fibrosis, neuroprotection or tissue-level functional benefit	Drug release; permeation; cytotoxicity; cell viability; uptake; inflammatory markers; apoptosis markers *in vitro*	Yes	In vitro release; cytotoxicity; cell viability; permeation; trabecular meshwork cells; retinal ganglion cells
Level 3	Conventional *in vivo* validation, usually short-term animal testing focused on delivery performance or IOP reduction	Studies were assigned to level 3 when animal experiments demonstrated ocular residence, tolerability, pharmacokinetics, short-term IOP lowering or basic *in vivo* biocompatibility, but lacked strong disease microenvironment modulation, long-term tissue integration or advanced translational endpoints	IOP reduction; ocular residence; pharmacokinetics; short-term tolerability; ocular irritation; basic histology	Yes	Rabbit model; rat model; IOP lowering; ocular retention; pharmacokinetics; *in vivo* tolerance
Level 4	Disease-relevant microenvironment intervention or functional tissue-engineering validation	Studies were assigned to level 4 when the intervention targeted glaucoma-specific pathological mechanisms or tissue interfaces, including post-surgical fibrosis, inflammatory or oxidative microenvironment modulation, trabecular meshwork modeling, RGC protection, outflow pathway reconstruction, or device-tissue interaction beyond simple delivery	Fibrosis suppression; RGC survival; outflow facility; trabecular meshwork function; inflammation control; tissue remodeling; disease-relevant histology	Yes	anti-Fibrosis; trabeculectomy; RGC neuroprotection; trabecular meshwork model; outflow pathway; tissue engineering
Level 5	Advanced translational evidence, long-term functional integration, large-animal validation or clinical evidence	Studies were assigned to level 5 when they provided long-term functional outcomes, clinical or human evidence, advanced translational device evaluation, sustained tissue integration, or disease-relevant functional recovery beyond short-term proof-of-concept	Long-term IOP control; clinical outcome; human study; large-animal validation; long-term implant performance; functional integration; sustained visual or neuroprotective outcome	Yes	Clinical trial; patient; long-term follow-up; large animal; sustained IOP control; implant performance
Review/Background	Review articles or background records used for contextual interpretation but not counted in level 1-5 core evidence statistics	Records were assigned to this category when they summarized drug delivery systems, biomaterials, glaucoma devices or related therapeutic strategies without presenting original experimental or clinical evidence eligible for level 1-5 coding	Narrative synthesis; systematic summary; technology overview; conceptual framework	No	review; mini-review; overview; drug delivery devices; nanotechnology review

Review/Background records were retained for contextual interpretation but excluded from Level 1-5 core evidence statistics.

### 2.6 Thematic classification of application scenarios

Each retained record was assigned to a primary application scenario according to title, abstract, keywords, and available metadata. The main themes included ocular or anterior chamber drug delivery, contact lens-based delivery system, *in situ* hydrogel, nano/microparticle composite system, anti-fibrosis after glaucoma surgery, glaucoma drainage device or minimally invasive glaucoma surgery-related material, trabecular meshwork model or tissue engineering, retinal ganglion cell neuroprotection or regeneration, 3D printing or bioprinting, and other or uncategorized.

Ocular or anterior chamber drug delivery was assigned to studies primarily focused on delivery of anti-glaucoma or intraocular pressure-lowering therapeutics to the ocular surface, anterior chamber, or intraocular tissues. Contact lens-based delivery system was assigned when the contact lens or lens material was the main platform. *In situ* hydrogel was assigned when sol-gel transition, thermosensitive behavior, pH-responsive gelation, or gel-forming formulation was central to the study. Nano/microparticle composite systems were assigned when nanoparticles, microparticles, liposomes, micelles, dendrimers, nanoemulsions, solid lipid nanoparticles, or related nano/microcarriers were the core functional component.

Anti-fibrosis after glaucoma surgery was assigned to studies targeting trabeculectomy, filtration surgery, bleb scarring, subconjunctival fibrosis, wound healing, or post-surgical scar modulation. Glaucoma drainage device or minimally invasive glaucoma surgery-related material was assigned to studies involving aqueous shunts, tube shunts, Ahmed valves, Baerveldt implants, Molteno implants, XEN gel stents, iStent, Hydrus, CyPass, PRESERFLO, or other glaucoma surgical devices, stents, coatings, or material interfaces.

Trabecular meshwork model or tissue engineering was assigned to studies focused on the trabecular meshwork, Schlemm canal, aqueous outflow pathway, organ-on-a-chip systems, microphysiological models, bioengineered outflow models, or tissue-engineered glaucoma-related platforms. Retinal ganglion cell neuroprotection or regeneration was assigned to studies involving retinal ganglion cell survival, optic nerve protection, axon regeneration, neurotrophic factor delivery, apoptosis inhibition, or neuroprotective material systems. 3D printing or bioprinting was assigned when additive manufacturing, printed scaffolds, printed implants, printed constructs, or biofabrication was a core technical component.

When more than one theme was present, the primary theme was assigned according to the dominant disease application and material function rather than isolated keyword occurrence. Ambiguous cases were manually reviewed. The theme classification rules are detailed in Table 3. The same harmonized theme framework was used for retained-record application-scenario mapping and core-evidence maturity analyses; review/background records were included in the retained-corpus distribution but excluded from core evidence-level and maturity analyses.

**TABLE 3:** Theme classification rules.

Theme	Inclusion criteria	Exclusion criteria	Typical materials or platforms	Typical disease endpoint
Ocular/anterior chamber drug delivery	Studies primarily designed to deliver antiglaucoma agents or IOP-lowering drugs to the ocular surface, anterior chamber or related ocular tissues	Exclude contact lens-specific systems, clearly defined *in situ* gel systems, GDD/MIGS device studies, and purely anti-fibrotic post-surgical studies unless drug delivery is the main purpose	Hydrogels; ocular inserts; intracameral depots; polymer carriers; sustained-release implants	Drug release; ocular residence; IOP reduction; pharmacokinetics
Contact lens-based delivery system	Studies in which contact lenses or lens-like materials were used as the main delivery or monitoring platform	Exclude general ocular drug delivery systems that do not use a contact lens or lens-based platform	Soft contact lenses; silicone hydrogel lenses; PHEMA lenses; medicated contact lenses	Drug release; corneal residence; IOP reduction; ocular compatibility
*In situ* hydrogel	Studies centered on sol-gel transition, thermoresponsive, pH-responsive or gel-forming ophthalmic formulations	Exclude conventional hydrogels without *in situ* gelation or responsive gel-forming behavior	Poloxamer; Pluronic; gellan gum; thermosensitive gels; pH-sensitive gels	Gelation; sustained release; ocular retention; IOP reduction
Nano/microparticle composite system	Studies using nanoparticles, microparticles, nanomicelles, liposomes, microspheres or related nano/micro-carriers for glaucoma-relevant delivery or intervention	Exclude purely bulk hydrogel or contact lens systems unless nano/micro-carriers are a core functional component	Nanoparticles; microparticles; liposomes; micelles; dendrimers; nanoemulsions; solid lipid nanoparticles	Controlled release; tissue penetration; IOP reduction; biocompatibility
Anti-fibrosis after glaucoma surgery	Studies targeting scarring, subconjunctival fibrosis, bleb failure or wound healing after trabeculectomy or glaucoma filtration surgery	Exclude general GDD/MIGS device material studies unless anti-fibrotic modulation is the primary endpoint	Anti-fibrotic hydrogels; drug-loaded depots; coatings; subconjunctival materials; mitomycin or 5-FU delivery systems	Fibrosis suppression; bleb survival; wound modulation; myofibroblast inhibition
GDD/MIGS-related material	Studies focusing on glaucoma drainage devices, aqueous shunts, tube shunts, stents or minimally invasive glaucoma surgery-related materials	Exclude post-trabeculectomy anti-fibrosis studies without device/stent/shunt involvement	Ahmed valve-related materials; Baerveldt-related materials; XEN gel stent; iStent; Hydrus; tube shunt coatings	Device patency; IOP control; tissue compatibility; long-term implant performance
Trabecular meshwork model/tissue engineering	Studies modeling, engineering or reconstructing trabecular meshwork, Schlemm canal, aqueous outflow pathway or related glaucoma tissue microenvironments	Exclude simple drug delivery studies mentioning trabecular meshwork only as a target tissue without model or tissue-engineering design	3D trabecular meshwork models; organ-on-a-chip; microfluidic systems; scaffolds; engineered outflow models	Outflow facility; tissue remodeling; disease modeling; tissue integration
RGC neuroprotection/regeneration	Studies targeting retinal ganglion cell survival, optic nerve protection, axon regeneration or neuroprotective glaucoma therapy using material-based systems	Exclude IOP-only studies without RGC, optic nerve or neuroprotective endpoints	Neuroprotective nanoparticles; hydrogels; scaffolds; stem-cell related platforms; sustained neurotrophic delivery systems	RGC survival; apoptosis inhibition; axon protection; optic nerve preservation
3D printing/bioprinting	Studies using 3D printing, bioprinting or additive manufacturing for glaucoma-relevant models, implants, scaffolds or delivery systems	Exclude non-printed scaffolds or hydrogels unless additive manufacturing is a central method	3D-printed constructs; bioprinted models; printed scaffolds; printed implants; microstructured devices	Model construction; tissue engineering; device customization; functional integration

Theme assignment was based on the primary disease application scenario rather than keyword frequency alone.

### 2.7 Translational feature annotation and theme maturity metrics

Each retained record was further annotated across three binary dimensions: smart material relevance, additive manufacturing relevance, and tissue-engineering relevance. Smart material relevance was assigned when the material system involved stimuli-responsive behavior, environmentally responsive release, sensing-related function, adaptive physicochemical behavior, or disease-microenvironment-responsive activity. Additive manufacturing relevance was assigned when 3D printing, bioprinting, additive manufacturing, printed scaffolds, printed implants, or process-structure design was central to the study. Tissue-engineering relevance was assigned when the record involved engineered tissues, scaffolds, bioengineered models, cell-supportive matrices, outflow-pathway models, retinal or optic nerve-related regenerative constructs, or functional tissue-like platforms.

Theme maturity was assessed within the core evidence set. For each theme, total record count, recent record count, recent share, mean evidence level, Level 4–5 record count and share, smart material relevance, additive manufacturing relevance, and tissue-engineering relevance were calculated. Recent growth was assessed using the ratio of records from 2021 to 2025 to records from 2006 to 2020, with a pseudo-count added to both periods to reduce instability in small themes.

### 2.8 Visualization and quality control

All analyses were descriptive. Data cleaning, classification checks, summary statistics, and visualization were performed using R software, version 4.5.3. Evidence-level distributions, theme-specific evidence structures, theme maturity metrics, and convergence among smart material, additive manufacturing, and tissue-engineering features were visualized according to the analytical aims of the study.

Quality control was applied at multiple stages. Metadata fields were checked after RIS parsing to identify incomplete or misaligned records. Duplicate removal was inspected using DOI and title information. Theme assignment and evidence-level classification were reviewed against predefined operational criteria. Ambiguous records and abbreviation-sensitive cases were manually audited. Final exclusions were made when records were unrelated to glaucoma-specific material intervention, when glaucoma was only incidentally mentioned, or when key terms were misinterpreted. Final quality-control exclusions were documented at the record level in Supplementary Table S2 to distinguish false-positive acronym matching, tangential ocular reviews, and non-material intervention records.

The classification framework was used as an operational evidence-mapping tool. It was not designed to replace full-text risk-of-bias assessment or clinical evidence grading systems. Evidence levels reflected the highest experimental or translational stage identifiable from the available bibliographic record and were used to compare structural maturity across themes. Country collaboration networks and institution-level analyses were not performed because the cleaned dataset did not contain sufficiently complete affiliation metadata for reliable geographic analysis.

## 3 Results

### 3.1 Corpus construction and analytical dataset

The study workflow and corpus construction process are summarized in Figure 1 and Table 1. Records were retrieved from Web of Science Core Collection, Scopus, and PubMed, followed by RIS-based parsing, metadata cleaning, duplicate removal, title and abstract screening, manual review, and final quality control.

The screening process began with 1,227 parsed records. After removal of 42 records before screening, 1,185 records entered title and abstract screening, of which 539 were excluded. The remaining 646 records underwent eligibility assessment. Among them, 599 records were directly included after first screening, whereas 47 records required manual review. Manual review excluded 46 records and added one record with clear glaucoma drug-delivery relevance despite insufficient abstract information. After final quality control, four additional records were excluded because they did not meet the glaucoma-specific material-intervention criteria. The final retained corpus included 596 records, consisting of 547 core evidence records and 49 review or background records. The full manual-review handling and final quality-control exclusions are listed in Supplementary Table S2.

The retained set and the core evidence set were used for different analytical purposes. The retained set was used for the overall literature landscape, application scenario distribution, and feature-overlap analysis. The core evidence set was used for evidence-level classification, theme-specific evidence distribution, and thematic maturity assessment.

### 3.2 Annual publication trends and evidence-stage composition

**FIGURE 2:**
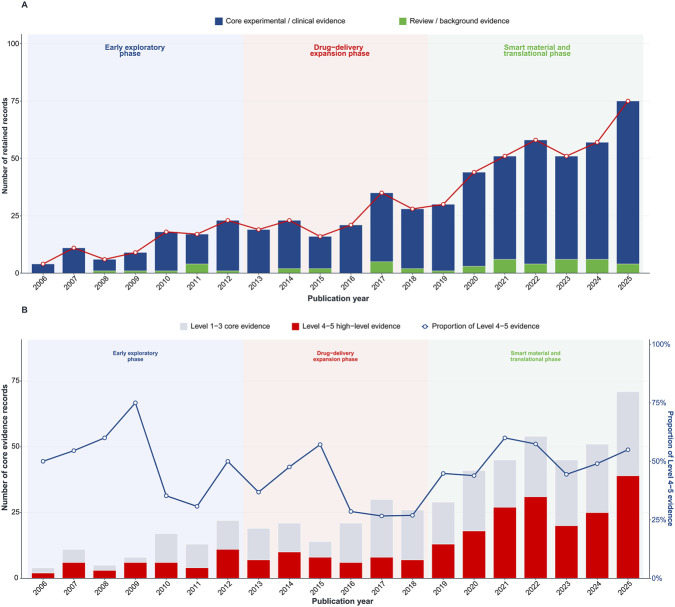
Annual publication trends and evidence-stage transition in glaucoma-related biomaterial research. **(A)** Annual distribution of retained records from 2006 to 2025, stratified by core evidence and review/background records. **(B)** Annual distribution of Level 1–3 and Level 4–5 core evidence records, with the line indicating the yearly proportion of Level 4–5 evidence.

Annual publication patterns are shown in Figure 2. The number of retained records increased across the study period from 2006 to 2025. Core evidence records formed the major part of the annual corpus, whereas review and background records represented a smaller component.

Figure 2 also displays the annual distribution of early-to-mid stage evidence and Level 4–5 evidence. Across the study period, Level 1–3 and Level 4–5 records were both present, with year-to-year variation in their relative proportions. The annual proportion of Level 4–5 evidence was not constant, showing that the evidence-stage composition changed over time rather than following a uniform pattern.

### 3.3 Disease application scenario distribution

**FIGURE 3:**
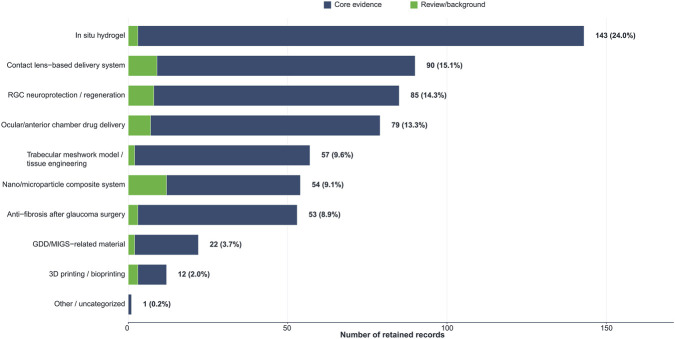
Distribution of disease application scenarios in the retained corpus. Horizontal bar plot showing the number and proportion of retained records assigned to each primary application scenario. Bars are stratified by core evidence and review/background records. GDD/MIGS denotes glaucoma drainage device/minimally invasive glaucoma surgery; RGC denotes retinal ganglion cell. Counts in Figure 3 were calculated from the retained corpus, including core evidence records and review/background records. Theme-specific evidence distributions and maturity metrics in Figures 5, 6 and Table 4 were calculated from the core evidence subset.

The distribution of application scenarios is shown in Figure 3. *In situ* hydrogel was the largest category in the retained corpus, with 143 records, accounting for 24.0% of all retained studies. Contact lens-based delivery system ranked second, with 90 records, accounting for 15.1%. RGC neuroprotection or regeneration included 85 records, accounting for 14.3%. Ocular or anterior chamber drug delivery included 79 records, accounting for 13.3%. Trabecular meshwork model or tissue engineering included 57 records, accounting for 9.6%. Nano/microparticle composite systems included 54 records, accounting for 9.1%.

Smaller categories included anti-fibrosis after glaucoma surgery, with 53 records, accounting for 8.9%; GDD/MIGS-related material, with 22 records, accounting for 3.7%; and 3D printing or bioprinting, with 12 records, accounting for 2.0%. Other or uncategorized records accounted for one record, or 0.2%.

For GDD/MIGS-related material, records were counted only when the title, abstract, keywords, or device context clearly referred to glaucoma drainage devices or minimally invasive glaucoma surgery. Records in which similar abbreviations referred to unrelated material terms were not included in this category.

### 3.4 Evidence-level architecture of core studies

**FIGURE 4:**
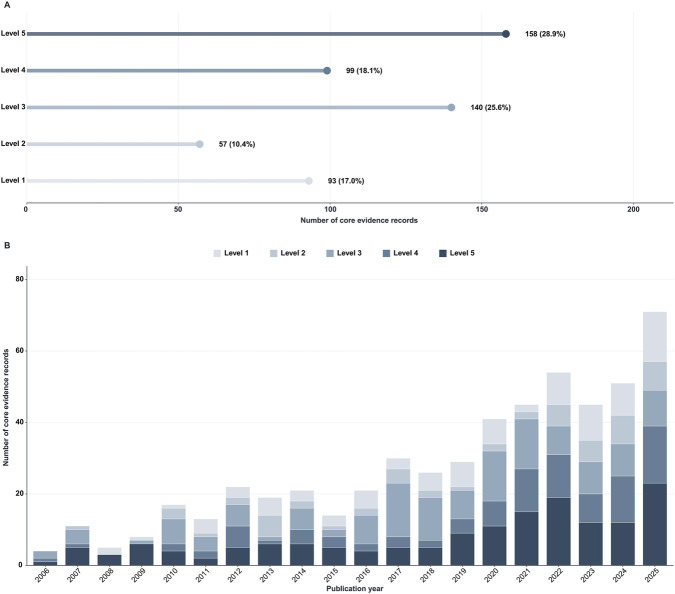
Evidence-level architecture of the core evidence set. **(A)** Overall distribution of Level 1–5 evidence among 547 core evidence records. **(B)** Annual stacked distribution of evidence levels from 2006 to 2025. Each record was assigned to the highest identifiable validation stage supported by its title, abstract, keywords, and available metadata; lower-level characterization or *in vitro* endpoints were not counted separately when higher-level validation was present. Evidence levels were used as operational categories for field-level mapping and should not be interpreted as formal clinical evidence grades.

The evidence-level composition of the 547 core evidence records is presented in Figure 4. Level 1 evidence included 93 records, Level 2 included 57 records, Level 3 included 140 records, Level 4 included 99 records, and Level 5 included 158 records.

When grouped by evidence stage, Level 1–3 records accounted for 290 records, or 53.0% of the core evidence set. Level 4–5 records accounted for 257 records, or 47.0%. Thus, the core evidence set contained comparable proportions of early-to-mid stage evidence and higher-level evidence, with a slight predominance of Level 1–3 records.

The annual evidence-level distribution is also shown in Figure 4. Material characterization, *in vitro* or cell-based validation, conventional animal validation, disease-microenvironment or tissue-engineering mechanism validation, and advanced translational or long-term evidence were all represented in the corpus, with different annual contributions across the study period.

### 3.5 Theme-specific evidence distribution

**FIGURE 5:**
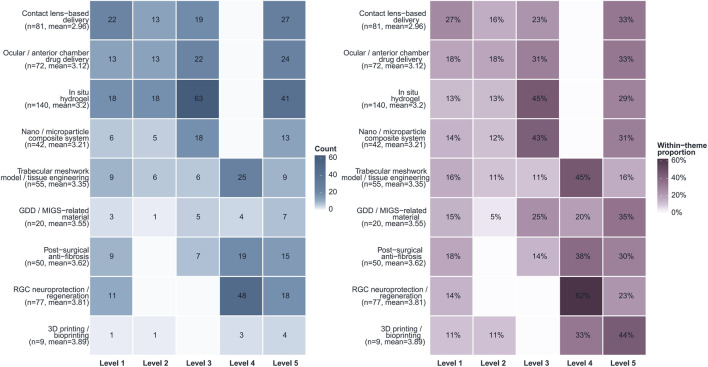
Theme-specific evidence-level distribution. Heatmaps showing the distribution of evidence levels across major application themes. The left panel presents record counts for each theme–evidence level combination. The right panel presents within-theme proportions. Blank cells indicate no records assigned to the corresponding evidence level. GDD/MIGS denotes glaucoma drainage device/minimally invasive glaucoma surgery.

Theme-specific evidence distributions are shown in Figure 5. The matrix presents the row-wise proportion of Level 1 to Level 5 evidence within each named theme. Other or uncategorized records were not emphasized in this figure.

RGC neuroprotection or regeneration had the highest Level 4 proportion among the named themes. Within this theme, Level 4 accounted for 62.3% and Level 5 accounted for 23.4%. Trabecular meshwork model or tissue engineering also showed a high proportion of Level 4 evidence, with Level 4 accounting for 45.5% and Level 5 accounting for 16.4%. Anti-fibrosis after glaucoma surgery included 38.0% Level 4 records and 30.0% Level 5 records.

3D printing or bioprinting was a small theme but had a high proportion of upper-level evidence in the matrix. Level 4 accounted for 33.3% and Level 5 accounted for 44.4%, while Level 3 accounted for 0.0%. GDD/MIGS-related material included 20.0% Level 4 records and 35.0% Level 5 records.

Delivery-centered themes showed broader evidence-level distributions. *In situ* hydrogel included 12.9% Level 1, 12.9% Level 2, 45.0% Level 3, 0.0% Level 4, and 29.3% Level 5 records. Nano/microparticle composite systems included 14.3% Level 1, 11.9% Level 2, 42.9% Level 3, 0.0% Level 4, and 31.0% Level 5 records. Ocular or anterior chamber drug delivery included 18.1% Level 1, 18.1% Level 2, 30.6% Level 3, 0.0% Level 4, and 33.3% Level 5 records. Contact lens-based delivery system included 27.2% Level 1, 16.0% Level 2, 23.5% Level 3, 0.0% Level 4, and 33.3% Level 5 records.

### 3.6 Theme maturity matrix

**FIGURE 6:**
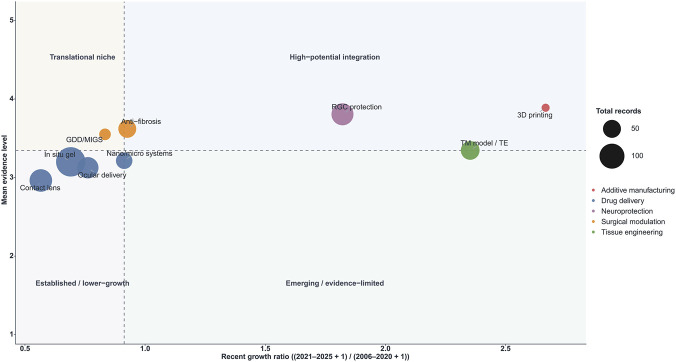
Theme maturity matrix of glaucoma-related biomaterial research. Bubble plot showing theme-level maturity based on recent growth ratio, mean evidence level, and total record count. The x-axis represents recent growth ratio, calculated as (records from 2021 to 2025 + 1)/(records from 2006 to 2020 + 1). The y-axis represents mean evidence level. Bubble size indicates total records, and color indicates theme type. GDD/MIGS denotes glaucoma drainage device/minimally invasive glaucoma surgery; TM/TE denotes trabecular meshwork/tissue engineering.

The theme maturity matrix is shown in Figure 6, and the numerical summary is provided in Table 4. The matrix used recent growth ratio as the x-axis, mean evidence level as the y-axis, and total record count as the bubble size. The quadrant labels in Figure 6 were used as interpretive categories based on relative positions in the maturity matrix and were not intended to represent formal clusters or statistically tested groups.

**TABLE 4:** Theme maturity summary.

Theme	Total records	Earlier records, 2006–2020	Recent records, 2021-2025	Recent share, 2021–2025	Recent growth ratio	Mean evidence level	Level 4-5 records	Level 4–5 share	Smart material share	Additive manufacturing share	Tissue engineering high-relevance share	Maturity classification
*In situ* hydrogel	140	83	57	40.7%	0.69	3.2	41	29.3%	27.9%	0.0%	0.0%	Established or lower-growth theme
Contact lens-based delivery system	81	52	29	35.8%	0.57	2.96	27	33.3%	4.9%	0.0%	1.2%	Established or lower-growth theme
RGC neuroprotection/regeneration	77	27	50	64.9%	1.82	3.81	66	85.7%	11.7%	1.3%	96.1%	High-potential integrated theme
Ocular/anterior chamber drug delivery	72	41	31	43.1%	0.76	3.12	24	33.3%	4.2%	1.4%	2.8%	Established or lower-growth theme
Trabecular meshwork model/tissue engineering	55	16	39	70.9%	2.35	3.35	34	61.8%	12.7%	1.8%	80.0%	High-potential integrated theme
Anti-fibrosis after glaucoma surgery	50	26	24	48.0%	0.93	3.62	34	68.0%	20.0%	0.0%	10.0%	Translational niche theme
Nano/microparticle composite system	42	22	20	47.6%	0.91	3.21	13	31.0%	0.0%	0.0%	0.0%	Established but heterogeneous delivery theme
GDD/MIGS-related material	20	11	9	45.0%	0.83	3.55	11	55.0%	25.0%	0.0%	5.0%	Translational niche theme
3D printing/bioprinting	9	2	7	77.8%	2.67	3.89	7	77.8%	22.2%	100.0%	100.0%	High-potential integrated theme

Recent share refers to the proportion of records published during 2021–2025 within each theme. Recent growth ratio was calculated as (records from 2021 to 2025 + 1)/(records from 2006 to 2020 + 1). This table summarizes named core evidence themes; one other/uncategorized core evidence record was not included in theme maturity interpretation.

Among the named core themes, *in situ* hydrogel remained the largest evidence base, with 140 records, but it showed a lower recent growth ratio and mean evidence level than several smaller disease-facing domains. RGC neuroprotection or regeneration included 77 core records and showed a high recent growth ratio, high mean evidence level, and the highest Level 4–5 share among the named themes. Trabecular meshwork model or tissue engineering included 55 core records and also showed strong recent expansion and a high Level 4–5 share. Anti-fibrosis after glaucoma surgery and GDD/MIGS-related material were smaller domains, but both showed relatively high mean evidence levels and substantial Level 4–5 proportions, supporting their interpretation as translational niche themes. 3D printing or bioprinting remained the smallest named theme, with nine core records, but showed the highest recent growth ratio and mean evidence level. Detailed numerical values are provided in Table 4.

Taken together, these results indicate that the largest themes were not necessarily the most translationally mature. *In situ* hydrogel formed the largest evidence base, whereas RGC neuroprotection or regeneration, trabecular meshwork model or tissue engineering, anti-fibrosis after glaucoma surgery, GDD/MIGS-related material, and 3D printing or bioprinting carried stronger disease-facing or integration-oriented evidence despite smaller record counts.

### 3.7 Material-application-endpoint pathway mapping

**FIGURE 7:**
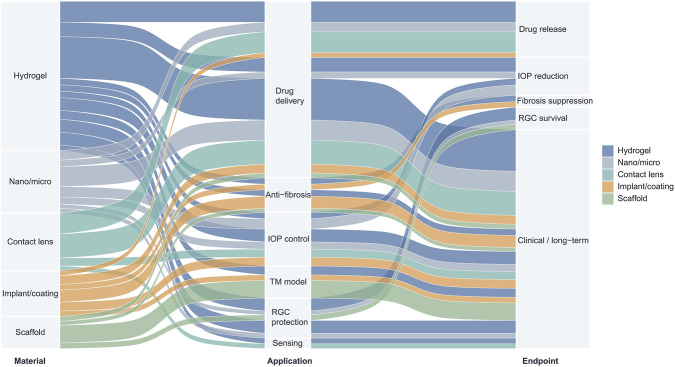
Material–application–endpoint pathway map. Sankey diagram showing dominant links from material categories to application scenarios and endpoints. The left layer represents material categories, the middle layer represents application scenarios, and the right layer represents endpoints. Flow width indicates the relative number of records within each pathway. IOP denotes intraocular pressure; RGC denotes retinal ganglion cell; TM denotes trabecular meshwork.

Figure 7 shows the pathway mapping from material categories to application scenarios and endpoints. The material layer included hydrogel, nano/micro systems, contact lens, implant or coating systems, and scaffold. The application layer included drug delivery, anti-fibrosis, IOP control, trabecular meshwork model, RGC protection, and sensing. The endpoint layer included drug release, IOP reduction, fibrosis suppression, RGC survival, and clinical or long-term outcomes.

The most prominent pathways originated from hydrogel and connected mainly with drug delivery, followed by endpoints related to drug release and IOP reduction. Nano/micro systems and contact lens platforms also contributed to delivery-centered pathways. Implant or coating systems were connected with anti-fibrosis, IOP control, and clinical or long-term endpoints. Scaffold-related pathways connected with trabecular meshwork model and RGC protection. In the endpoint layer, drug release, IOP reduction, fibrosis suppression, RGC survival, and clinical or long-term outcomes were all represented. This pathway map was used as a descriptive summary of material-application-endpoint relationships and was not used as the primary basis for theme maturity interpretation.

### 3.8 Convergence of smart material, additive manufacturing, and tissue-engineering relevance

**FIGURE 8:**
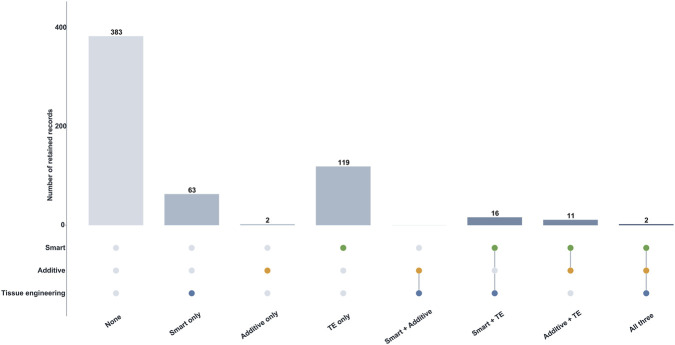
Convergence of smart material, additive manufacturing, and tissue-engineering relevance. UpSet-style plot showing overlap among three annotated features: smart material relevance, additive manufacturing relevance, and tissue-engineering relevance. Bars represent the number of retained records in each feature combination. TE denotes tissue engineering.

The overlap among smart material relevance, additive manufacturing relevance, and tissue-engineering relevance is shown in Figure 8. Among all retained records, 383 records, or 64.3%, were classified as none of the three dimensions. Tissue-engineering relevance alone accounted for 119 records, or 20.0%. Smart material relevance alone accounted for 63 records, or 10.6%.

Combined feature categories were less frequent. Smart material plus tissue-engineering relevance accounted for 16 records, or 2.7%. Additive manufacturing plus tissue-engineering relevance accounted for 11 records, or 1.8%. Additive manufacturing alone accounted for 2 records, or 0.3%. The all-three category also included 2 records, or 0.3%. No record was classified as smart material plus additive manufacturing without tissue-engineering relevance. Overall, most records did not show convergence across the three dimensions, and fully integrated smart material, additive manufacturing, and tissue-engineering relevance remained rare.

## 4 Discussion

This study reconstructs glaucoma-related biomaterial research as an evidence architecture rather than a publication landscape. The main finding is not that the field lacks advanced evidence. In the core evidence set, Level 1–3 studies accounted for 53.0%, whereas Level 4–5 studies accounted for 47.0%. The field therefore contains a substantial body of disease-relevant, long-term, translational, or clinical evidence. The more important pattern is that this evidence is unevenly distributed. Large delivery-oriented categories, including *in situ* hydrogels, contact lens-based delivery system, ocular or anterior chamber drug delivery, and nano/microparticle platforms, formed the broad base of the literature. Smaller domains, including retinal ganglion cell protection, trabecular meshwork modeling and tissue engineering, anti-fibrosis after glaucoma surgery, GDD/MIGS-related material interfaces, and 3D printing or bioprinting, carried a greater proportion of disease-facing or integration-oriented evidence. This imbalance is the central structural feature of the field.

The dominance of delivery-centered systems is understandable. Glaucoma treatment still depends heavily on long-term intraocular pressure control, and conventional topical therapy is limited by tear clearance, low ocular bioavailability, frequent dosing, and poor adherence (Almidani et al., 2026). Hydrogels, contact lenses, nano/microcarriers, and sustained-release systems directly address these barriers. In this sense, drug delivery remains the technical foundation of glaucoma biomaterials (Huang et al., 2024). However, the evidence map also shows why delivery volume should not be mistaken for translational maturity. Many delivery studies remain centered on formulation, loading efficiency, release kinetics, cytocompatibility, ocular residence, or short-term pressure reduction. These endpoints are necessary, but they do not necessarily demonstrate disease modification, tissue-level function, or long-term therapeutic integration. Glaucoma is shaped not only by drug exposure, but also by aqueous outflow dysfunction, fibrotic wound healing, neural vulnerability, inflammatory stress, and material–tissue interface responses (Garg et al., 2019; Clement Freiberg et al., 2022; Mansberger et al., 2022; Berdahl et al., 2024; Tsai et al., 2025). A material platform that improves residence time is therefore not equivalent to a system that alters the biological course of the disease.

The more disease-specific parts of the evidence architecture appear in smaller but functionally sharper domains. Retinal ganglion cell protection is one such domain. Because vision loss in glaucoma ultimately reflects retinal ganglion cell and optic nerve damage, material systems that deliver neuroprotective agents, regulate apoptosis, support axonal integrity, or modulate neuroinflammatory stress occupy a different translational position from pressure-lowering delivery platforms (D'Angelo et al., 2024). These studies are more directly tied to neural survival and disease progression. Trabecular meshwork modeling and tissue engineering form another important domain. The trabecular meshwork and Schlemm canal regulate aqueous outflow, but their cellular phenotype, extracellular matrix organization, stiffness, and flow-responsive behavior are difficult to reproduce in conventional culture systems (Stamer et al., 2015; Feng et al., 2025). Engineered outflow models, scaffold-based systems, and microphysiological platforms therefore provide a route to connect material design with glaucoma mechanism research. These platforms are especially relevant to functional tissue engineering because they ask whether engineered constructs can reproduce disease-relevant behavior, not merely whether a material is biocompatible.

Surgical-material studies define a third disease-facing branch. Fibrosis remains a major cause of failure after glaucoma filtration surgery, and conventional anti-metabolite strategies are constrained by toxicity, wound-healing complications, and limited spatiotemporal control (Shao et al., 2023; Zhang et al., 2025). Anti-fibrotic biomaterials may allow more localized and sustained modulation of fibroblast activation, extracellular matrix deposition, inflammation, and scar formation. Similarly, GDD/MIGS-related materials involve a different set of engineering problems from ordinary drug delivery. Glaucoma drainage devices and minimally invasive glaucoma surgery platforms introduce artificial interfaces into small and reactive ocular tissues. Their performance depends on biointegration, surface response, fibrosis, inflammation, fluid stability, and long-term patency (Dorairaj et al., 2022). These questions place material design at the interface between surgery, tissue response, and device function.

The relationship between glaucoma biomaterials and the scope of smart materials, additive manufacturing, and functional tissue engineering is still incomplete. The literature contains responsive hydrogels, controlled-release systems, engineered outflow models, scaffold-based constructs, printed platforms, and neuroprotective materials. Yet these elements often appear in separate technical trajectories. Records integrating smart material relevance, additive manufacturing relevance, and tissue-engineering relevance were rare. This pattern suggests that the field has accumulated many useful components, but has not yet routinely assembled them into unified systems that combine environmental responsiveness, manufacturable architecture, and disease-relevant functional validation. This distinction is important for positioning glaucoma biomaterials within smart materials and functional tissue-engineering research. In our annotation, smart material relevance was not assigned simply because a record involved a hydrogel, nanoparticle, contact lens, or implantable material; it required stimuli-responsive behavior, sensing-related function, adaptive physicochemical behavior, environmentally responsive release, or disease-microenvironment-responsive activity. A glaucoma biomaterial should not be considered “smart” only because it responds to pH, temperature, light, enzymes, or redox conditions *in vitro*. Its responsiveness should be linked to a glaucoma-relevant trigger and a measurable biological output, such as fibrosis suppression, outflow restoration, inflammatory control, RGC survival, or long-term implant compatibility (Iliadis et al., 2025; Lee et al., 2025).

Additive manufacturing also requires a stricter interpretation. Printing alone does not establish tissue-engineering relevance. For glaucoma applications, the value of additive manufacturing depends on whether it improves spatial organization, construct reproducibility, cell or matrix placement, mechanical stability, outflow-related function, or implant-interface design (Wlodarczyk-Biegun et al., 2022; Abukunna et al., 2025). This distinction matters because ocular structures are small, geometrically constrained, and functionally sensitive to fluid movement, pressure gradients, and neural integrity. Future studies should therefore report not only printability or shape fidelity, but also process–structure–function relationships, including printing parameters, crosslinking strategy, degradation behavior, mechanical properties, cell distribution, and disease-relevant functional readouts. Without these elements, additive manufacturing risks remaining a fabrication label rather than a translational strategy.

The present findings also point to a reporting problem. Many studies describe materials, carriers, or responses, but do not consistently connect them to disease-specific endpoints. For delivery studies, longer release should be linked to sustained pharmacodynamic benefit or long-term IOP control. For anti-fibrotic materials, release profiles should be connected to wound-healing modulation, fibroblast behavior, extracellular matrix remodeling, and tissue compatibility. For trabecular meshwork models, construct design should be connected to outflow resistance, pressure response, cytoskeletal organization, extracellular matrix remodeling, and disease-like dysfunction. For RGC-related systems, material performance should be connected to cell survival, axonal preservation, apoptosis suppression, trophic support, or retinal and optic nerve function (Chitranshi et al., 2019; Vigneswara and Ahmed, 2019; Pham et al., 2022). These reporting standards would make the field more comparable and would also improve its readiness for AI-assisted evidence synthesis, material optimization, and translational prioritization.

Several limitations should be considered. First, classification was based on titles, abstracts, keywords, and available metadata. Information present only in full texts may have been missed, especially details on follow-up duration, material mechanism, tissue integration, or functional endpoints. Second, the five-level evidence framework was an operational classification system designed for field-level mapping. It should not be interpreted as a formal clinical evidence hierarchy or risk-of-bias assessment. Third, theme assignment required boundary decisions. Drug delivery studies mentioning the trabecular meshwork were not classified as trabecular meshwork modeling or tissue engineering unless model construction or outflow-pathway engineering was central to the study. GDD/MIGS-related records were also restricted to clear surgical-device or material-interface contexts. Fourth, review and background records were retained for contextual mapping but excluded from core evidence-level statistics. Fifth, country and institution analyses were not performed because affiliation metadata were not sufficiently complete or standardized after cleaning.

## 5 Conclusion

In conclusion, glaucoma-related biomaterial research has moved beyond a purely delivery-centered field, but its transition toward smart, manufacturable, and function-oriented tissue-engineering systems remains uneven. Traditional delivery platforms remain the largest evidence base, whereas RGC protection, trabecular meshwork modeling, anti-fibrotic surgical modulation, GDD/MIGS-related materials, and 3D printing represent smaller but more disease-facing directions. Future work should place greater emphasis on disease-relevant endpoints, reproducible material design, functional tissue models, engineered surgical interfaces, and long-term validation and long-term functional or translational validation.

## Data Availability

The original contributions presented in the study are included in the article/Supplementary Material, further inquiries can be directed to the corresponding author.
