# Supplementary material for: Evidence architecture of glaucoma-related biomaterials reveals an uneven transition toward smart materials, additive manufacturing, and functional tissue engineering
Source: Front Bioeng Biotechnol. 2026 Jun 12;14:1868297. doi: 10.3389/fbioe.2026.1868297 (PMC13303748; doi:10.3389/fbioe.2026.1868297)
Supplement: Supplementary file 1 [file Table1.docx]

**Supplementary Tables**

**Supplementary Table S1. Full search strategy for Web of Science, Scopus, and PubMed**

| **Database** | **Search set** | **Exact search strategy** | **Limits and field settings** | **Export and integration** |
| --- | --- | --- | --- | --- |
| Web of Science Core Collection | Main search | TS=((glaucoma* OR "ocular hypertension" OR "intraocular pressure" OR "trabecular meshwork" OR "retinal ganglion cell*" OR RGC* OR "optic nerve" OR "glaucoma filtration surgery" OR trabeculectomy OR "glaucoma drainage device*" OR "minimally invasive glaucoma surgery" OR MIGS) AND (hydrogel* OR "in situ gel*" OR "gel-forming" OR "thermosensitive gel*" OR thermoresponsive OR "stimuli-responsive" OR "smart material*" OR biomaterial* OR polymer* OR scaffold* OR coating* OR implant* OR "contact lens*" OR "drug delivery" OR "controlled release" OR "sustained release" OR nanoparticle* OR microparticle* OR nanocarrier* OR liposome* OR micelle* OR "3D printing" OR bioprint* OR "additive manufacturing" OR "tissue engineering")) | Publication years: 2006-2025. Search field: Topic. Document types and language were not restricted at retrieval; eligibility was assessed during screening. | Exported to EndNote-compatible format, then merged and deduplicated in EndNote 21. |
| Web of Science Core Collection | Sensitivity search | TS=((glaucoma* OR "ocular hypertension" OR "intraocular pressure" OR "trabecular meshwork" OR "retinal ganglion cell*" OR RGC* OR "optic nerve" OR "glaucoma filtration surgery" OR trabeculectomy OR "glaucoma drainage device*" OR "minimally invasive glaucoma surgery" OR MIGS) AND ("smart material*" OR "responsive material*" OR "stimuli-responsive" OR thermoresponsive OR "ROS-responsive" OR "pH-responsive" OR "3D print*" OR bioprint* OR "additive manufacturing" OR "organ-on-a-chip" OR microphysiological OR "trabecular meshwork model" OR "tissue engineering" OR scaffold*)) | Publication years: 2006-2025. Used to capture smart/responsive materials, 3D printing, additive manufacturing, organ-on-chip and tissue-engineering records. | Merged with main search records before deduplication. |
| Scopus | Main search | TITLE-ABS-KEY((glaucoma* OR "ocular hypertension" OR "intraocular pressure" OR "trabecular meshwork" OR "retinal ganglion cell*" OR RGC* OR "optic nerve" OR "glaucoma filtration surgery" OR trabeculectomy OR "glaucoma drainage device*" OR "minimally invasive glaucoma surgery" OR MIGS) AND (hydrogel* OR "in situ gel*" OR "gel-forming" OR "thermosensitive gel*" OR thermoresponsive OR "stimuli-responsive" OR "smart material*" OR biomaterial* OR polymer* OR scaffold* OR coating* OR implant* OR "contact lens*" OR "drug delivery" OR "controlled release" OR "sustained release" OR nanoparticle* OR microparticle* OR nanocarrier* OR liposome* OR micelle* OR "3D printing" OR bioprint* OR "additive manufacturing" OR "tissue engineering")) AND PUBYEAR > 2005 AND PUBYEAR < 2026 | Publication years: 2006-2025. Search field: TITLE-ABS-KEY. Document types and language were not restricted at retrieval; eligibility was assessed during screening. | Exported as RIS and merged in EndNote 21. |
| Scopus | Sensitivity search | TITLE-ABS-KEY((glaucoma* OR "ocular hypertension" OR "intraocular pressure" OR "trabecular meshwork" OR "retinal ganglion cell*" OR RGC* OR "optic nerve" OR "glaucoma filtration surgery" OR trabeculectomy OR "glaucoma drainage device*" OR "minimally invasive glaucoma surgery" OR MIGS) AND ("smart material*" OR "responsive material*" OR "stimuli-responsive" OR thermoresponsive OR "ROS-responsive" OR "pH-responsive" OR "3D print*" OR bioprint* OR "additive manufacturing" OR "organ-on-a-chip" OR microphysiological OR "trabecular meshwork model" OR "tissue engineering" OR scaffold*)) AND PUBYEAR > 2005 AND PUBYEAR < 2026 | Publication years: 2006-2025. Used to capture smart/responsive materials, 3D printing, additive manufacturing, organ-on-chip and tissue-engineering records. | Merged with main search records before deduplication. |
| PubMed | Main search | (glaucoma*[Title/Abstract] OR "ocular hypertension"[Title/Abstract] OR "intraocular pressure"[Title/Abstract] OR "trabecular meshwork"[Title/Abstract] OR "retinal ganglion cell*"[Title/Abstract] OR RGC*[Title/Abstract] OR "optic nerve"[Title/Abstract] OR "glaucoma filtration surgery"[Title/Abstract] OR trabeculectomy[Title/Abstract] OR "glaucoma drainage device*"[Title/Abstract] OR "minimally invasive glaucoma surgery"[Title/Abstract] OR MIGS[Title/Abstract]) AND (hydrogel*[Title/Abstract] OR "in situ gel*"[Title/Abstract] OR "gel-forming"[Title/Abstract] OR "thermosensitive gel*"[Title/Abstract] OR thermoresponsive[Title/Abstract] OR "stimuli-responsive"[Title/Abstract] OR "smart material*"[Title/Abstract] OR biomaterial*[Title/Abstract] OR polymer*[Title/Abstract] OR scaffold*[Title/Abstract] OR coating*[Title/Abstract] OR implant*[Title/Abstract] OR "contact lens*"[Title/Abstract] OR "drug delivery"[Title/Abstract] OR "controlled release"[Title/Abstract] OR "sustained release"[Title/Abstract] OR nanoparticle*[Title/Abstract] OR microparticle*[Title/Abstract] OR nanocarrier*[Title/Abstract] OR liposome*[Title/Abstract] OR micelle*[Title/Abstract] OR "3D printing"[Title/Abstract] OR bioprint*[Title/Abstract] OR "additive manufacturing"[Title/Abstract] OR "tissue engineering"[Title/Abstract]) AND ("2006/01/01"[Date - Publication] : "2025/12/31"[Date - Publication]) | Publication dates: 2006/01/01 to 2025/12/31. Search field: Title/Abstract. | Exported as NBIB and imported into EndNote 21. |
| PubMed | Sensitivity search | (glaucoma*[Title/Abstract] OR "ocular hypertension"[Title/Abstract] OR "intraocular pressure"[Title/Abstract] OR "trabecular meshwork"[Title/Abstract] OR "retinal ganglion cell*"[Title/Abstract] OR RGC*[Title/Abstract] OR "optic nerve"[Title/Abstract] OR "glaucoma filtration surgery"[Title/Abstract] OR trabeculectomy[Title/Abstract] OR "glaucoma drainage device*"[Title/Abstract] OR "minimally invasive glaucoma surgery"[Title/Abstract] OR MIGS[Title/Abstract]) AND ("smart material*"[Title/Abstract] OR "responsive material*"[Title/Abstract] OR "stimuli-responsive"[Title/Abstract] OR thermoresponsive[Title/Abstract] OR "ROS-responsive"[Title/Abstract] OR "pH-responsive"[Title/Abstract] OR "3D print*"[Title/Abstract] OR bioprint*[Title/Abstract] OR "additive manufacturing"[Title/Abstract] OR "organ-on-a-chip"[Title/Abstract] OR microphysiological[Title/Abstract] OR "trabecular meshwork model"[Title/Abstract] OR "tissue engineering"[Title/Abstract] OR scaffold*[Title/Abstract]) AND ("2006/01/01"[Date - Publication] : "2025/12/31"[Date - Publication]) | Publication dates: 2006/01/01 to 2025/12/31. Used to capture smart/responsive materials, 3D printing, additive manufacturing, organ-on-chip and tissue-engineering records. | Merged with main search records before deduplication. |

*Note. The search strategy is reported as the full reproducible logic used for cross-database retrieval.*

**Supplementary Table S2. Manual-review handling and final quality-control exclusions**

**Panel A. Manual-review and final quality-control decision rules**

| **Stage** | **Trigger or record type** | **Decision rule and action** | **Number of records** |
| --- | --- | --- | --- |
| Manual-review pool | Ambiguous title/abstract screening result, missing abstract, or broad ocular material/drug-delivery review with possible glaucoma relevance. | Inspect title, abstract, keywords, document type and glaucoma-specific disease context. Retain only records with direct glaucoma/ocular hypertension/IOP context and material, device, drug-delivery, tissue-engineering, anti-fibrosis, RGC or trabecular-meshwork relevance. | 47 |
| Manual-review inclusion | Missing abstract but direct glaucoma-specific scope was evident from title/keywords. | Retained as review/background when the title and keywords clearly focused on glaucoma drug delivery devices. | 1 |
| Manual-review exclusion: broad ocular review | Broad ocular drug-delivery or material review with glaucoma mentioned only as one of several ocular diseases or drug examples. | Excluded from the main retained set to prevent non-glaucoma-specific reviews from inflating the evidence map. | 36 |
| Manual-review exclusion: insufficient evidence | Missing abstract or title-only record without enough evidence for a glaucoma-specific material intervention. | Excluded unless the title and keywords directly established glaucoma-specific material/device relevance. | 10 |
| Final quality control | Records retained after screening but found to be false-positive, tangential or not material-intervention studies after final inspection. | Excluded after QC and documented at record level. | 4 |

*Note. Manual review was used for records that could not be safely resolved using automated title/abstract screening alone. One record was retained as review/background; all other manual-review records were excluded from the final retained set.*

**Panel B. Record-level manual-review outcomes**

| **ID** | **Year** | **Title** | **Journal** | **Manual-review trigger** | **Final decision** | **Final reason** |
| --- | --- | --- | --- | --- | --- | --- |
| 966 | 2008 | Recent patents on ocular drug delivery systems | Recent Patents on Drug Delivery and Formulation | Broad ocular material/drug-delivery review with possible but not central glaucoma relevance. | Excluded after manual review | Broad ocular material/drug-delivery review; glaucoma was not the central disease context. |
| 112 | 2010 | Biotechnological Production and Application of Hyaluronan | Biopolymers | Missing abstract; cannot safely screen by title alone. | Excluded after manual review | Missing abstract and title/metadata were insufficient to establish direct glaucoma-specific material relevance. |
| 956 | 2010 | Recent advances in ophthalmic drug delivery | Therapeutic Delivery | Broad ocular material/drug-delivery review with possible but not central glaucoma relevance. | Excluded after manual review | Broad ocular material/drug-delivery review; glaucoma was not the central disease context. |
| 1196 | 2011 | An updated patent review on ocular drug delivery systems with potential for commercial viability | Recent Patents on Drug Delivery and Formulation | Broad ocular material/drug-delivery review with possible but not central glaucoma relevance. | Excluded after manual review | Broad ocular material/drug-delivery review; glaucoma was not the central disease context. |
| 585 | 2011 | In situ gelling system and other possible innovative approach for ocular disease: A review | Research Journal of Pharmacy and Technology | Broad ocular material/drug-delivery review with possible but not central glaucoma relevance. | Excluded after manual review | Broad ocular material/drug-delivery review; glaucoma was not the central disease context. |
| 670 | 2011 | Intravitreal intrusion of an intrascleral MIRAgel buckling implant | Japanese Journal of Ophthalmology | Missing abstract; cannot safely screen by title alone. | Excluded after manual review | Missing abstract and title/metadata were insufficient to establish direct glaucoma-specific material relevance. |
| 25 | 2012 | Advances in hydrogels applied to degenerative diseases | Current Pharmaceutical Design | Broad ocular material/drug-delivery review with possible but not central glaucoma relevance. | Excluded after manual review | Broad ocular material/drug-delivery review; glaucoma was not the central disease context. |
| 395 | 2012 | Environment-sensitive polymers for ophthalmic drug delivery | Journal of Drug Delivery Science and Technology | Broad ocular material/drug-delivery review with possible but not central glaucoma relevance. | Excluded after manual review | Broad ocular material/drug-delivery review; glaucoma was not the central disease context. |
| 766 | 2012 | Nanomaterials for Ocular Drug Delivery | Macromolecular Bioscience | Broad ocular material/drug-delivery review with possible but not central glaucoma relevance. | Excluded after manual review | Broad ocular material/drug-delivery review; glaucoma was not the central disease context. |
| 190 | 2013 | Contact lenses: Promising devices for ocular drug delivery | Journal of Ocular Pharmacology and Therapeutics | Broad ocular material/drug-delivery review with possible but not central glaucoma relevance. | Excluded after manual review | Broad ocular material/drug-delivery review; glaucoma was not the central disease context. |
| 611 | 2016 | Infiltration of hydrogel implant into the sclera with calcification: a case report with histologic findings | Canadian Journal of Ophthalmology | Missing abstract; cannot safely screen by title alone. | Excluded after manual review | Missing abstract and title/metadata were insufficient to establish direct glaucoma-specific material relevance. |
| 305 | 2017 | Drug delivery to the eye: What benefits do nanocarriers offer? | Nanomedicine | Broad ocular material/drug-delivery review with possible but not central glaucoma relevance. | Excluded after manual review | Broad ocular material/drug-delivery review; glaucoma was not the central disease context. |
| 803 | 2017 | Novel Glaucoma Drug Delivery Devices | International Ophthalmology Clinics | Missing abstract; cannot safely screen by title alone. | Retained as review/background | Title and keywords directly indicated glaucoma drug delivery devices despite missing abstract. |
| 998 | 2018 | A review on in situ gel forming ophthalmic drug delivery systems | Research Journal of Pharmacy and Technology | Broad ocular material/drug-delivery review with possible but not central glaucoma relevance. | Excluded after manual review | Broad ocular material/drug-delivery review; glaucoma was not the central disease context. |
| 964 | 2018 | Recent Innovations in Drug Delivery for Retinal Diseases | Advances in Ophthalmology and Optometry | Missing abstract; cannot safely screen by title alone. | Excluded after manual review | Missing abstract and title/metadata were insufficient to establish direct glaucoma-specific material relevance. |
| 754 | 2019 | Nano-based drug delivery system: Recent strategies for the treatment of ocular disease and future perspective | Recent Patents on Drug Delivery and Formulation | Broad ocular material/drug-delivery review with possible but not central glaucoma relevance. | Excluded after manual review | Broad ocular material/drug-delivery review; glaucoma was not the central disease context. |
| 762 | 2019 | Nanoformulations for ocular delivery of drugs - A patent perspective | Recent Patents on Drug Delivery and Formulation | Broad ocular material/drug-delivery review with possible but not central glaucoma relevance. | Excluded after manual review | Broad ocular material/drug-delivery review; glaucoma was not the central disease context. |
| 862 | 2019 | Orthokeratology for Managing Myopia Progression in Children | Advances in Ophthalmology and Optometry | Missing abstract; cannot safely screen by title alone. | Excluded after manual review | Missing abstract and title/metadata were insufficient to establish direct glaucoma-specific material relevance. |
| 879 | 2019 | Pharmaceutical challenges and perspectives in developing ophthalmic drug formulations | Journal of Pharmaceutical Investigation | Broad ocular material/drug-delivery review with possible but not central glaucoma relevance. | Excluded after manual review | Broad ocular material/drug-delivery review; glaucoma was not the central disease context. |
| 972 | 2019 | Reconsidering the central role of mucins in dry eye and ocular surface diseases | Progress in Retinal and Eye Research | Broad ocular material/drug-delivery review with possible but not central glaucoma relevance. | Excluded after manual review | Broad ocular material/drug-delivery review; glaucoma was not the central disease context. |
| 747 | 2020 | Mucoadhesive Micro-/Nano Carriers in Ophthalmic Drug Delivery: an Overview | BioNanoScience | Broad ocular material/drug-delivery review with possible but not central glaucoma relevance. | Excluded after manual review | Broad ocular material/drug-delivery review; glaucoma was not the central disease context. |
| 813 | 2020 | Novel Polyvinyl Pyrrolidone-Loaded Olopatadine HCl-Laden Doughnut Contact Lens to Treat Allergic Conjunctivitis | Journal of Pharmaceutical Sciences | Missing abstract; cannot safely screen by title alone. | Excluded after manual review | Missing abstract and title/metadata were insufficient to establish direct glaucoma-specific material relevance. |
| 829 | 2020 | Ocular drug delivery system: Challenges and approaches | International Journal of Applied Pharmaceutics | Broad ocular material/drug-delivery review with possible but not central glaucoma relevance. | Excluded after manual review | Broad ocular material/drug-delivery review; glaucoma was not the central disease context. |
| 907 | 2020 | Polysaccharide as renewable responsive biopolymer for in situ gel in the delivery of drug through ocular route | International Journal of Biological Macromolecules | Broad ocular material/drug-delivery review with possible but not central glaucoma relevance. | Excluded after manual review | Broad ocular material/drug-delivery review; glaucoma was not the central disease context. |
| 832 | 2021 | Ocular drug delivery systems: A review | Farmacia | Broad ocular material/drug-delivery review with possible but not central glaucoma relevance. | Excluded after manual review | Broad ocular material/drug-delivery review; glaucoma was not the central disease context. |
| 985 | 2021 | Research progress of nanostructured lipid carriers in ocular drug delivery | Drug Delivery Letters | Broad ocular material/drug-delivery review with possible but not central glaucoma relevance. | Excluded after manual review | Broad ocular material/drug-delivery review; glaucoma was not the central disease context. |
| 1123 | 2021 | Ten years of knowledge of nano-carrier based drug delivery systems in ophthalmology: Current evidence, challenges, and future prospective | International Journal of Nanomedicine | Broad ocular material/drug-delivery review with possible but not central glaucoma relevance. | Excluded after manual review | Broad ocular material/drug-delivery review; glaucoma was not the central disease context. |
| 1169 | 2021 | Topical timolol 0.5% gel-forming solution for erythema in rosacea: A quantitative, split-face, randomized, and rater-masked pilot clinical trial | Journal of the American Academy of Dermatology | Missing abstract; cannot safely screen by title alone. | Excluded after manual review | Missing abstract and title/metadata were insufficient to establish direct glaucoma-specific material relevance. |
| 80 | 2022 | Bibliometric analysis of articles on nanoemulsion and/or in-situ gel for ocular drug delivery system published during the 2011-2021 period | Pharmacia | Broad ocular material/drug-delivery review with possible but not central glaucoma relevance. | Excluded after manual review | Broad ocular material/drug-delivery review; glaucoma was not the central disease context. |
| 217 | 2022 | Current and future therapeutic strategies for the treatment of retinal neurodegenerative diseases | Neural Regeneration Research | Missing abstract; cannot safely screen by title alone. | Excluded after manual review | Missing abstract and title/metadata were insufficient to establish direct glaucoma-specific material relevance. |
| 797 | 2022 | NOVEL APPROACHES IN OCULAR DRUG DELIVERY-A REVOLUTION | International Journal of Applied Pharmaceutics | Broad ocular material/drug-delivery review with possible but not central glaucoma relevance. | Excluded after manual review | Broad ocular material/drug-delivery review; glaucoma was not the central disease context. |
| 969 | 2022 | Recent progress in the use of thermogelling polymers for treatment of ophthalmic conditions | Progress in Biomedical Engineering | Broad ocular material/drug-delivery review with possible but not central glaucoma relevance. | Excluded after manual review | Broad ocular material/drug-delivery review; glaucoma was not the central disease context. |
| 981 | 2022 | Removal of MIRAgel Scleral Buckle Implants: The Direct Aspiration Technique | Retina | Missing abstract; cannot safely screen by title alone. | Excluded after manual review | Missing abstract and title/metadata were insufficient to establish direct glaucoma-specific material relevance. |
| 1203 | 2022 | The Use of Polymer Blends in the Treatment of Ocular Diseases | Pharmaceutics | Broad ocular material/drug-delivery review with possible but not central glaucoma relevance. | Excluded after manual review | Broad ocular material/drug-delivery review; glaucoma was not the central disease context. |
| 130 | 2023 | Carbohydrate polymer-based bioadhesive formulations and their potentials for the treatment of ocular diseases: A review | International Journal of Biological Macromolecules | Broad ocular material/drug-delivery review with possible but not central glaucoma relevance. | Excluded after manual review | Broad ocular material/drug-delivery review; glaucoma was not the central disease context. |
| 438 | 2023 | Eyes on New Product Development | Journal of Ocular Pharmacology and Therapeutics | Missing abstract; cannot safely screen by title alone. | Excluded after manual review | Missing abstract and title/metadata were insufficient to establish direct glaucoma-specific material relevance. |
| 755 | 2023 | Nano-based eye drop: Topical and noninvasive therapy for ocular diseases | Advanced Drug Delivery Reviews | Broad ocular material/drug-delivery review with possible but not central glaucoma relevance. | Excluded after manual review | Broad ocular material/drug-delivery review; glaucoma was not the central disease context. |
| 765 | 2023 | Nanomaterial-based ophthalmic drug delivery | Advanced Drug Delivery Reviews | Broad ocular material/drug-delivery review with possible but not central glaucoma relevance. | Excluded after manual review | Broad ocular material/drug-delivery review; glaucoma was not the central disease context. |
| 825 | 2023 | Ocular Delivery of Therapeutic Agents by Cell-Penetrating Peptides | Cells | Broad ocular material/drug-delivery review with possible but not central glaucoma relevance. | Excluded after manual review | Broad ocular material/drug-delivery review; glaucoma was not the central disease context. |
| 834 | 2023 | Ocular Drug Delivery: a Comprehensive Review | AAPS PharmSciTech | Broad ocular material/drug-delivery review with possible but not central glaucoma relevance. | Excluded after manual review | Broad ocular material/drug-delivery review; glaucoma was not the central disease context. |
| 864 | 2023 | Overcoming Treatment Challenges in Posterior Segment Diseases with Biodegradable Nano-Based Drug Delivery Systems | Pharmaceutics | Broad ocular material/drug-delivery review with possible but not central glaucoma relevance. | Excluded after manual review | Broad ocular material/drug-delivery review; glaucoma was not the central disease context. |
| 914 | 2023 | Precision Medicines for Retinal Lipid Metabolism-Related Pathologies | Journal of Personalized Medicine | Broad ocular material/drug-delivery review with possible but not central glaucoma relevance. | Excluded after manual review | Broad ocular material/drug-delivery review; glaucoma was not the central disease context. |
| 960 | 2023 | Recent Advances of Ocular Drug Delivery Systems: Prominence of Ocular Implants for Chronic Eye Diseases | Pharmaceutics | Broad ocular material/drug-delivery review with possible but not central glaucoma relevance. | Excluded after manual review | Broad ocular material/drug-delivery review; glaucoma was not the central disease context. |
| 1197 | 2023 | Updates on Biodegradable Formulations for Ocular Drug Delivery | Pharmaceutics | Broad ocular material/drug-delivery review with possible but not central glaucoma relevance. | Excluded after manual review | Broad ocular material/drug-delivery review; glaucoma was not the central disease context. |
| 1000 | 2025 | A Review on Ocular Nanoformulation Based Formulations with Highlights on Pediatric Ocular Pharmacokinetics | Pharmaceutical Nanotechnology | Broad ocular material/drug-delivery review with possible but not central glaucoma relevance. | Excluded after manual review | Broad ocular material/drug-delivery review; glaucoma was not the central disease context. |
| 149 | 2025 | Chemically modified hyaluronic acid derivatives as ocular drug carriers: A review | International Journal of Biological Macromolecules | Broad ocular material/drug-delivery review with possible but not central glaucoma relevance. | Excluded after manual review | Broad ocular material/drug-delivery review; glaucoma was not the central disease context. |
| 831 | 2025 | Ocular drug delivery systems based on nanotechnology: a comprehensive review for the treatment of eye diseases | Discover Nano | Broad ocular material/drug-delivery review with possible but not central glaucoma relevance. | Excluded after manual review | Broad ocular material/drug-delivery review; glaucoma was not the central disease context. |

*Note. The retained manual-review record was Novel Glaucoma Drug Delivery Devices (ID 803), because the title and keywords directly supported glaucoma-specific drug-delivery relevance despite the absence of an abstract.*

**Panel C. Final quality-control exclusions**

| **ID** | **Year** | **Title** | **Journal** | **QC category** | **Reason for exclusion** |
| --- | --- | --- | --- | --- | --- |
| 1122 | 2011 | Temperature-sensitive molecularly imprinted microgels with esterase activity | Science China-Chemistry | Not glaucoma/MIGS; false positive from MIGs acronym | Exclude. MIGs in this record means molecularly imprinted microgels, not minimally invasive glaucoma surgery; no glaucoma-specific disease context. |
| 819 | 2010 | Numerical Simulations of Ethacrynic Acid Transport from Precorneal Region to Trabecular Meshwork | Annals of Biomedical Engineering | Computational ocular drug transport model | Exclude from core material evidence analysis. It is a numerical transport model for ethacrynic acid, without a hydrogel/biomaterial/device intervention. |
| 63 | 2022 | Application of Vitamin E TPGS in ocular therapeutics - Attributes beyond excipient | Journal of the Indian Chemical Society | Broad ocular therapeutics review | Exclude from core analysis. Broad Vitamin E TPGS ocular therapeutics review; glaucoma appears as one of several diseases, not a glaucoma-focused material study. |
| 561 | 2022 | Immune modulating nanoparticles for the treatment of ocular diseases | Journal of Nanobiotechnology | Broad ocular nanoparticle immunomodulation review | Exclude from core analysis. Broad ocular disease nanoparticle review; not sufficiently glaucoma-specific for the main dataset. |

*Note. These four records were removed after final QC to prevent false-positive MIGS matching, non-material computational modeling, and tangential broad ocular reviews from entering the retained mapping dataset.*
